# A diagnostic model of nerve root compression localization in lower lumbar disc herniation based on random forest algorithm and surface electromyography

**DOI:** 10.3389/fnhum.2023.1176001

**Published:** 2023-07-04

**Authors:** Hujun Wang, Yingpeng Wang, Yingqi Li, Congxiao Wang, Shuyan Qie

**Affiliations:** Department of Rehabilitation, Beijing Rehabilitation Hospital, Capital Medical University, Beijing, China

**Keywords:** lumbar disc herniation, surface electromyography, random forest, diagnosis model, nerve root compression

## Abstract

**Objective:**

This study aimed to investigate the muscle activation of patients with lumbar disc herniation (LDH) during walking by surface electromyography (SEMG) and establish a diagnostic model based on SEMG parameters using random forest (RF) algorithm for localization diagnosis of compressed nerve root in LDH patients.

**Methods:**

Fifty-eight patients with LDH and thirty healthy subjects were recruited. The SEMG of tibialis anterior (TA) and lateral gastrocnemius (LG) were collected bilaterally during walking. The peak root mean square (RMS-peak), RMS-peak time, mean power frequency (MPF), and median frequency (MF) were analyzed. A diagnostic model based on SEMG parameters using RF algorithm was established to locate compressed nerve root, and repeated reservation experiments were conducted for verification. The study evaluated the diagnostic efficiency of the model using accuracy, precision, recall rate, F1-score, Kappa value, and area under the receiver operating characteristic (ROC) curve.

**Results:**

The results showed that delayed activation of TA and decreased activation of LG were observed in the L5 group, while decreased activation of LG and earlier activation of LG were observed in the S1 group. The RF model based on eight SEMG parameters showed an average accuracy of 84%, with an area under the ROC curve of 0.93. The RMS peak time of TA was identified as the most important SEMG parameter.

**Conclusion:**

These findings suggest that the RF model can assist in the localization diagnosis of compressed nerve roots in LDH patients, and the SEMG parameters can provide further references for optimizing the diagnosis model in the future.

## 1. Introduction

Lumbar disc herniation (LDH) is a common cause of low back pain and lower limb neuralgia (Furlan et al., 2009). The highest incidence of LDH occurs at the L4/5 and L5/S1 levels, with most patients experiencing radiculopathy involving a single nerve root, typically the L5 or S1 root (Al-Khawaja et al., 2016).

At present, clinical symptoms, physical examination and imaging findings are usually combined to determine the diagnosis of LDH and the corresponding nerve root compression. However, LDH is a disease in which physical examination, symptoms and imaging findings are not always reliable or correlated. Previous studies have found that the ability to diagnose radiculopathy caused by LDH is not ideal either by physical examination alone or by isolated imaging findings. Magnetic resonance imaging (MRI) is the gold standard to evaluate the structural relationship between intervertebral disc, surrounding soft tissue and nerve tissue (Li et al., 2015). However, MRI is usually performed in the resting state, which cannot monitor and evaluate the compression and functional states during movement. Several studies have found that many people without neurological symptoms exhibit positive MRI signs (Brinjikji et al., 2015a), and the accuracy of MRI in the diagnosis of compressed nerve roots is also lower (Lee and Lee, 2012; Brinjikji et al., 2015b; de Schepper et al., 2016). Therefore, there is a need to explore other means to support the localization diagnosis of LDH from a functional perspective.

Surface electromyography (SEMG) is a prevalent tool for functional assessment, enabling real-time, quantitative evaluation, and analysis of an individual’s dynamic neuromuscular function (Wakeling, 2009). Prior research has illuminated the neuromuscular function alterations following LDH from various perspectives. LDH patients typically exhibit diminished muscle strength and endurance, weakness in certain lower limb muscles, and fatigue in lumbar muscles (Dedering, 2012; Supuk et al., 2014; Djordjevic et al., 2015). Moreover, studies have posited that LDH patients with differing nerve root compressions display distinct EMG characteristics (Li et al., 2018). Our study aimed to delve further into the application of SEMG parameters in diagnosing LDH patients. However, additional exploration is required to analyze synchronous gait and SEMG changes, summarize muscle activation patterns, and more accurately classify and identify these patterns in patients with different nerve root compression. In this study, we concentrated on the tibialis anterior (TA) and lateral gastrocnemius (LG) muscles, because these muscles exhibited significant alterations in their SEMG characteristics in LDH patients with L5 and S1 nerve root compression. In instances of L5 nerve root compression, neurological control disorders are primarily observed in the TA. Conversely, in cases of S1 nerve root compression, these disorders are predominantly exhibited by the LG (Li et al., 2018). These observations suggest that when a specific nerve root is compressed, the functional state of the muscles primarily innervated by that nerve root changes.

Machine learning, a novel data processing method, can extract valuable information from vast amounts of data through learning and training, and construct effective prediction models. Random forest (RF) is a potent method that has found extensive application in the medical field. Machine learning has been employed in gait recognition, robot rehabilitation, motion control, among other fields (He et al., 2020; Shi et al., 2020). Therefore, our aim is to analyze the SEMG characteristics of LDH patients with different compressed nerve roots, summarize muscle activation regularity, establish an RF diagnostic model, and verify its diagnostic efficiency. By doing so, we aspire to provide a fresh approach for the localization diagnosis of LDH.

## 2. Materials and methods

### 2.1. Participants

A total of 58 patients with LDH scheduled for lumbar decompression were recruited for this study from the Beijing Rehabilitation Hospital, Capital Medical University in Beijing, China. There were 29 patients with L4/5 herniation combined with L5 nerve root compression (L5 group), and 29 patients with L5/S1 herniation combined with S1 nerve root compression (S1 group). Thirty healthy adults (Healthy group) without previous neurological or musculoskeletal diseases or surgery were recruited as a control group. The sample size was preliminary estimated using G*Power 3.1 software.^1^ Based on the results of a pilot study, the effect size was set at 1.06, the significance level at two-tailed α = 0.05, and the statistical power at 0.95, which indicated that a sample size of 24 was required. Considering the possible dropout rates and other uncertainties, 30 participants were planned for each group. However, one participant in each patient group was unable to complete the experiment due to personal reasons. Inclusion criteria for patients with LDH: (1) patients with a confirmed diagnosis of LDH with sciatic radicular pain; (2) herniated disc segments requiring MRI and surgical confirmation; (3) compressed nerve roots limited to L5 or S1 nerve roots; (4) indications for surgery and the need for surgical treatment; and (5) no contraindication to neurophysiology and can undergo SEMG. LDH patients with the following symptoms were excluded: (1) pacemaker or any other metal implant in the body; (2) related or other peripheral nerve diseases and abnormal motor fiber conduction; (3) spastic paralysis or other muscle diseases of lower limb muscles, such as cerebral palsy or muscular dystrophy; (4) previous history of spinal surgery; (5) clinical manifestations of lumbar spinal stenosis; and (6) combined with other serious diseases, such as severe cardiopulmonary disease, defined as a condition that requires continuous oxygen therapy or hospitalization for respiratory failure.

Healthy controls with the following symptoms were excluded: (1) abnormal gait due to congenital skeletal deformity or neurological disorders, such as cerebral palsy or multiple sclerosis; (2) lower extremity degenerative diseases and clinical symptoms, such as osteoarthritis or peripheral arterial disease, which affect walking function; (3) pregnant or perinatal women; and (4) suffering from other diseases that affect walking and daily activities, such as severe heart failure or end-stage renal disease.

**FIGURE 1:**
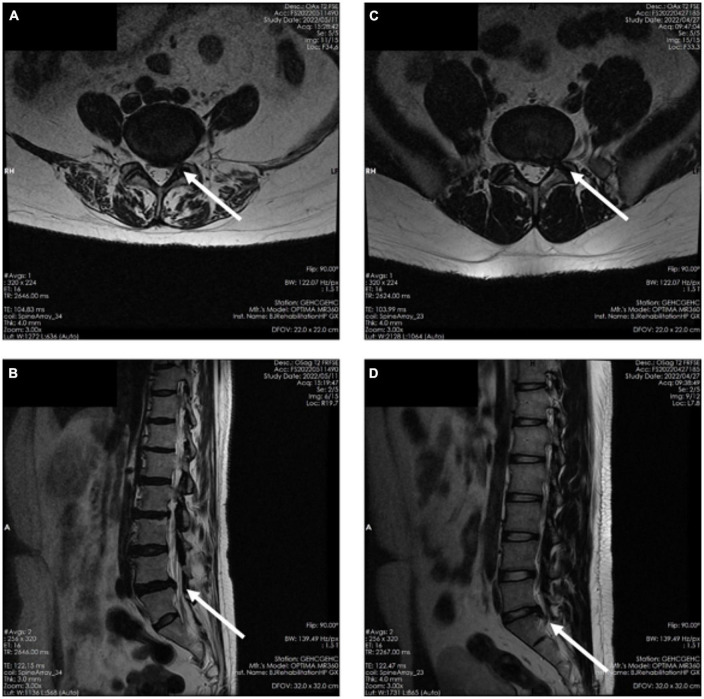
**(A)** Magnetic resonance imaging axial planes in patients with L4/L5 nerve root compression. **(B)** MRI sagittal planes in patients with L4/L5 nerve root compression. **(C)** MRI axial planes in patients with L5/S1 nerve root compression. **(D)** MRI sagittal planes in patients with L5/S1 nerve root compression planes.

Visual Analogue Scale (VAS) Pain scores and Japanese Orthopaedic Association scores (JOA) were obtained for all patients and the general characteristics of all subjects are shown in Table 1. Although MRI has some diagnostic limitations and does not guarantee 100% diagnostic accuracy, in current clinical practice it is still the primary basis for the localized diagnosis of nerve root compression in LDH. In the present study, patients were initially enrolled by physician examination, special examination, clinical symptoms, and MRI diagnosis, subjected to SEMG testing and further confirmed by surgery (Gurdjian et al., 1961; Al Nezari et al., 2013). MRI of a typical patient with nerve root compression is shown in Figure 1. No participants had neuroelectrophysiological contraindication. All subjects signed informed consent, and the study was approved by the Ethics Committee of Beijing Rehabilitation Hospital, Capital Medical University, Beijing, China.

**TABLE 1:** General characteristics of subjects.

	L5 group	S1 group	Healthy group
Number of people	29	29	30
Male: female	18:11	18:11	19:11
Age (years)	39.90 ± 15.18	41.86 ± 13.36	37.40 ± 14.70
Height (cm)	171.79 ± 10.30	169.59 ± 8.72	170.90 ± 8.88
Body weight (kg)	77.38 ± 16.10	70.69 ± 8.96	70.07 ± 16.44
BMI	26.09 ± 4.11	24.56 ± 2.24	23.87 ± 4.59
Leg pain (VAS)^#^	8 (5, 9)	7 (6, 8)	/
Low back pain (VAS)^#^	1 (2, 6)	1 (0, 3)	/
JOA score^#^	12 (6.5, 15)	13 (10, 14)	/

^#^Data does not follow normal distribution and is expressed as median (interquartile range).

### 2.2. SEMG measurement

#### 2.2.1. Instrument

**FIGURE 2:**
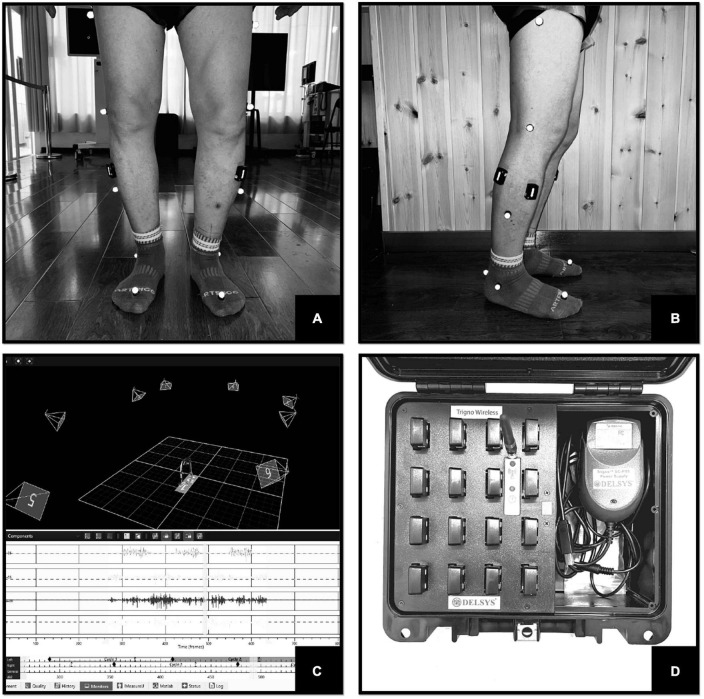
**(A)** Frontal view of the test. **(B)** Lateral view of the test. **(C)** Gait and SEMG capture interface. **(D)** Trigno™ Wireless Systems.

Surface electromyography signals were measured by DELSYS wireless dynamic EMG tester (Trigno™ Wireless Systems, Delsys Inc., USA, Figure 2D). The electrodes were placed on the surface of the muscle belly. Its sampling frequency is up to 2,000 Hz, transmission range is 20 m, and it can detect up to 16 muscles at the same time. The SEMG signal was synchronized with an 8-camera 3D motion capture system (Vicon, Oxford, UK) and two embedded force platforms (AMTI, Watertown, MA, USA) to divide gait cycles (Figure 2C). The gait cycle was defined using a heel strike frame on the force platform. The Vicon system and force measurement platform had sampling frequencies of 100 and 1,000 Hz, respectively. The Vicon system used a Plug-in gait model with 16 markers to define the body segments.

#### 2.2.2. Measurement methods

The tests in this study were conducted in a dedicated room with a clean, distraction-free environment. Participants wore close-fitting, non-black, and non-reflective clothing to minimize capture errors. Prior to testing, the skin of the bilateral TA and LG muscles was cleaned and prepared. According to the guidelines established by the Surface ElectroMyoGraphy for the Non-Invasive Assessment of Muscles (SENIAM) project,^2^ the collection electrodes of the Delsys wireless dynamic EMG tester were strategically positioned on the most prominent portions of TA and LG muscles on both sides (Figures 2A, B). The EMG signals were filtered using a band-pass filter with a range of 20–500 Hz. Subjects walked at their own comfortable speed until at least six successful trials were captured (excluding the initial acceleration and deceleration phases of the assessment).

#### 2.2.3. Parameters and data analysis

At the end of the test, the synchronization results of the Vicon and DELSYS data were imported into Python software for data processing and analysis. The SEMG parameters included in this study are as follows: (1) Time domain parameters: root mean square peak (RMS-peak) and RMS-peak time (i.e., the onset of the RMS-peak within the gait cycle). The RMS-peak represents the muscle force exerted during exercise; the RMS-peak time reflects the time of muscle activation during the gait cycle (Merletti et al., 2008). The specific data were processed as follows: RMS values during each gait cycle were calculated using a 30 ms window and a step length of 20 ms (Merletti et al., 2009). After normalizing the time according to the gait cycle, the average RMS value of the six gait cycles is calculated and the distribution of the RMS values is plotted, and then the RMS-peak and RMS-peak times (as a percentage of the gait cycle) are calculated. (2) Frequency domain parameters: the mean power frequency (MPF) and median frequency (MF) were chosen to reflect mainly the degree of muscle fatigue (Molinari et al., 2006). The specific data processing methods are as follows. First, the fast Fourier transform of the SEMG signal was performed to calculate the MPF and MF for each gait cycle, and then the average MPF and MF for the six gait cycles were calculated.

### 2.3. Establishment of RF diagnosis model

#### 2.3.1. Method of model establishment

To establish the diagnostic model, we employed the RF algorithm using Python 3.7 scikit learn. To mitigate individual differences, we selected parameters that showed significant differences between the two lower limbs. Specifically, we considered the absolute value of the difference between the parameters of healthy controls and the difference between symptomatic and asymptomatic sides of LDH patients.

#### 2.3.2. Process of model establishment

(1) Architecture of input and output layers: the RF model used in this study comprises eight input parameters and three output layers: no compression, L5 nerve root compression, and S1 nerve root compression. The input parameters consist of the SEMG parameters of TA and LG, namely RMS-peak, RMS-peak time, MPF, and MF.
(2) Training parameter settings: (1) Sample size setting: we selected 88 subjects, with 50% of them being allocated to the training set and the remaining 50% to the prediction set. (2) Superparameter setting: we selected n_Estimators, which refers to the number of sub-datasets generated by bootstrapping the original dataset, and set it as 50 in this study. (3) During the training process, all data were used in each round. We set the stopping criteria based on two situations: firstly, when the required accuracy is achieved (RMS error reaches 0.005), the training is stopped. Secondly, when the training process fails to achieve the required accuracy, we stop the training until the maximum number of iterations, which is set to 1,500 times, is reached.

#### 2.3.3. Model validation

During the experiments, the accuracy, precision, recall rate, F1-score, and Kappa values were calculated 10 times using the repeated reservation experiment principle. Additionally, the area under the receiver operating characteristic (ROC) curve was utilized to evaluate the efficiency of the diagnosis model.

#### 2.3.4. Establishment of the final model

In order to ensure the reliability of the diagnosis results, the testing procedure of the RF diagnosis model was repeated up to 10 times. If the results were not satisfactory, the procedure was repeated starting from the screening of independent variables. However, if the results were deemed satisfactory and reliable, the data from all patients were used to retrain and establish the RF diagnosis model. This approach aimed to ensure that the final diagnosis model had high accuracy, precision, recall rate, F1-score, Kappa values, and efficiency in detecting the different levels of nerve root compression.

### 2.4. Statistical analysis

The data were presented as mean ± standard error or median (interquartile range) based on the distribution characteristics. Normal distribution of data was assessed using the K-S test. Paired *t*-test and one-way ANOVA were used for comparison between groups for normally distributed data. For non-normally distributed data, non-parametric rank sum test such as Wilcoxon-Mann-Whitney test was used for comparison of two related groups, Kruskal-Wallis rank sum test was used for comparison of multiple groups, and pairwise comparison of multiple groups was performed using Bonferroni test. Statistical analysis was performed using SPSS 26.0 and *P* < 0.05 was considered statistically significant.

## 3. Results

### 3.1. SEMG characteristics of patients with different nerve root compression

**FIGURE 3:**
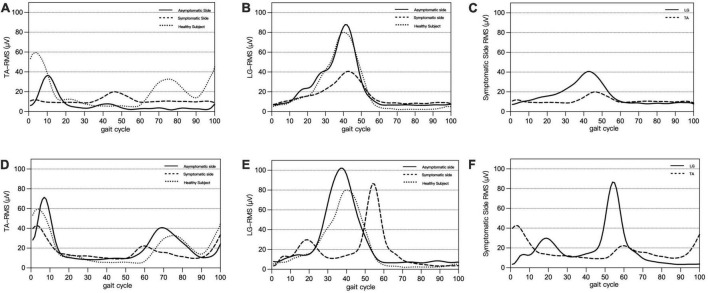
Panels **(A–C)** are typical SEMG presentations of L5 nerve root compression: on the symptomatic side, delayed activation of TA **(A)** and decreased peak RMS of LG **(B)**, showing co-contraction of TA and LG **(C)**. Panels **(D–F)** are typical SEMG presentations of S1 nerve root compression: on the symptomatic side, activation of the LG is shifted forward **(D)**, peak RMS of the TA is decreased **(E)**, and a double peak and co-contraction of the LG and TA is found **(F)**.

In the healthy group, the SEMG performance of each healthy subject was combined and averaged for their left and right sides, as all healthy subjects walked symmetrically. Their peak RMS, RMS-peak time, MPF, and MF were not significantly different from the asymptomatic side of patients in the L5 and S1 groups (Figure 3).

In L5 group, compared with the asymptomatic side, the RMS-peak time of TA in the symptomatic side was significantly delayed (*P* < 0.001), the MPF and MF of TA were significantly decreased (both *P* < 0.001); the RMS-peak of LG was also significantly decreased (*P* = 0.016) (Table 2). The delayed activation of TA was manifested by a later occurrence in the gait cycle [symptomatic side: 35 (25.5, 61), asymptomatic side 11 (7.5, 15), *P* < 0.001] and resulted in the co-contraction with LG (Figure 3).

**TABLE 2:** Surface electromyography characteristics of patients with L5 nerve root compression during walking.

		Symptomatic side	Asymptomatic side	*P*-value
		**Median (interquartile range)/** **mean ± SE**	**Median (interquartile range)/** **mean ± SE**	
TA	RMS-peak (μV)^#^	48.89 (19.26, 69.17)	34.16 (26.15, 100.16)	0.256
	RMS-peak time (%)^#^	35 (25.5, 61)	11 (7.5, 15)	<0.001*
	MPF (Hz)	62.74 ± 4.28	77.55 ± 5.58	<0.001*
	MF (Hz)^#^	50.33 (38.75, 64.71)	75.69 (64.57, 94.36)	<0.001*
LG	RMS-peak (μV)^#^	24.99 (13.89, 35.38)	30.12 (25.27, 40.97)	0.016*
	RMS-peak time (%)^#^	43 (31.5, 45.5)	43 (39.5, 48)	0.741
	MPF (Hz)^#^	82.67 (58.36, 98.70)	96.28 (40.77, 106.21)	0.614
	MF (Hz)	68.19 ± 4.69	70.67 ± 5.02	0.122

*P < 0.05.

^#^Data does not follow normal distribution and is expressed as median (interquartile range).

In S1 group, compared with the asymptomatic side, as for LG, the RMS-peak in the symptomatic side was significantly decreased (*P* = 0.003), the RMS-peak time was significantly moved forward (*P* < 0.001), the MPF and MF were significantly decreased (both *P* < 0.001), and the RMS-peak of TA in the symptomatic side was also significantly decreased (*P* = 0.043) (Table 3). The early activation of LG was manifested by an earlier occurrence in the gait cycle [affected side: 27 (14.5, 37.5), the contralateral side: 44 (38.5, 46.5), *P* < 0.001], showing a trend of bimodal activation and co-contraction with TA (Figure 3).

**TABLE 3:** Surface electromyography characteristics of patients with S1 nerve root compression during walking.

		Symptomatic side	Asymptomatic side	*P*-value
		**Median (interquartile range)/** **mean ± SE**	**Median (interquartile range)/** **mean ± SE**	
TA	RMS-peak (μ V)^#^	39.63 (35.49, 66.34)	62.49 (33.72, 156.43)	0.003*
	RMS-peak time (%)^#^	6 (4, 8)	5 (4, 6.5)	0.082
	MPF (Hz)^#^	66.67 (54.96, 81.12)	87.03 (62.77, 97.46)	0.071
	MF (Hz)^#^	54.22 (39.39, 65.42)	60.67 (44.84, 77.80)	0.239
LG	RMS-peak (μ V)	45.10 ± 4.80	62.28 ± 7.01	0.043*
	RMS-peak time (%)^#^	27 (14.5, 37.5)	44 (38.5, 46.5)	<0.001*
	MPF (Hz)	69.14 ± 4.24	95.34 ± 3.59	<0.001*
	MF (Hz)	54.15 ± 4.49	80.39 ± 5.15	<0.001*

*P < 0.05.

^#^Data does not follow normal distribution and is expressed as median (interquartile range).

Compared to the healthy group (Table 4), the L5 group showed significant delays in the RMS-peak time of TA (*P* < 0.001) and significant decreases in the MF (*P* = 0.002) and MPF (*P* = 0.001) of TA and LG. Similarly, the S1 group showed significant differences in the RMS-peak (*P* = 0.043) and MPF (*P* < 0.001) of LG and in the MPF (*P* = 0.033) and MF (*P* = 0.001) of TA, when compared to the healthy group. Additionally, the RMS-peak time of TA was significantly delayed in the L5 group compared to the S1 group (*P* < 0.001), while the RMS-peak of LG was significantly decreased (*P* = 0.001). Conversely, the RMS-peak time of LG was significantly earlier in the S1 group than in the L5 group (*P* = 0.045).

**TABLE 4:** Surface electromyography characteristics between LDH patients and healthy subjects during walking.

		L5 group	S1 group	Healthy group	*P*-value
		**Median (interquartile range) / mean ± SE**	**Median (interquartile range) / mean ±SE**	**Median (interquartile range) / mean ± SE**	
TA	RMS-peak (μ V)^#^	47.43 (22.49, 80.25)	54.84 (36.83, 97.89)	45.31 (29.34, 74.50)	0.303
	RMS-peak time (%)^#^	23.50 (17, 38)^✩ Δ^	6 (4, 8.5)	5.25 (2.38, 9.13)	<0.001*
	MPF (Hz)	70.15 ± 4.79	78.71 ± 5.06^✩^	63.29 ± 5.26	0.101
	MF (Hz)	61.96 ± 3.83^✩^	60.32 ± 3.98^✩^	83.26 ± 5.78	0.001*
LG	RMS-peak (μ V)^#^	28.74 (20.31, 41.24)^Δ^	54.27 (31.09, 70.31)^✩^	30.99 (23.42, 55.37)	0.002*
	RMS-peak time (%)^#^	42 (26, 50.5)	34 (29.25, 41.5)^Δ^	38.75 (23.88, 43)	0.313
	MPF (Hz)	79.03 ± 5.00^✩^	82.24 ± 3.39^✩^	58.93 ± 3.96	<0.001*
	MF (Hz)	69.43 ± 2.89	67.26 ± 4.16	77.59 ± 4.27	0.096

^#^Data does not follow normal distribution.

*There were significant differences among three groups (*P* < 0.05).

^✩^There was significant difference either L5 group or S1 group compared with healthy group (*P* < 0.05).

^Δ^There was significantly different between L5 and S1 group (*P* < 0.05).

### 3.2. Establishment of RF diagnosis model based on SEMG parameters

In this study, we selected the difference of parameters between the bilateral lower limbs as the input parameter. According to our statistical results, there were significant differences in the RMS-peak and RMS-peak time of TA, as well as the RMS-peak, RMS-peak time, and MPF of LG when compared to the healthy group. Furthermore, when compared with the patients in the L5 and S1 groups, significant differences were observed in the bilateral RMS-peak and RMS-peak time of TA (Table 5).

**TABLE 5:** Differences of bilateral SEMG parameters in three group.

	L5 group	S1 group	Healthy group	*P*-value
TA-RMS peak^#^	13.19 (6.28, 22.84)^Δ✩^	21.90 (17.57, 30.59)^✩^	8.06 (4.43, 11.33)	<0.001*
TA-RMS-peak time^#^	26 (14, 44.5) ^Δ✩^	1 (0.5, 4)	1 (0, 4)	<0.001*
TA-MPF^#^	15.42 (5.41, 28.89)	12.02 (5.78, 20.75)	11.97 (5.58, 29.91)	0.567
TA-MF^#^	10.91 (6.26, 32.24)	9.71 (3.92, 17.65)	13.0 (7.08, 25.80)	0.487
LG-RMS peak^#^	9.93 (4.50, 16.68)^✩^	12.83 (4.34, 31.31)^✩^	5.16 (1.84, 9.28)	0.001*
LG-RMS-peak time^#^	20 (3, 34.5)	29.69 (15.34, 51.22)^✩^	1.5 (1, 31.25)	<0.001*
LG-MPF^#^	25.09 (4.78, 56.82)^✩^	23.21 (16.73, 46.32)^✩^	7.85 (3.86, 11.35)	<0.001*
LG-MF^#^	27.21 (9.27, 58.21)	18 (10, 29)	13.26 (10.17, 31.14)	0.188

^#^Data does not follow normal distribution.

*There were significant differences among three groups (*P* < 0.05).

^✩^There was significant difference either L5 group or S1 group compared with healthy group (*P* < 0.05).

^Δ^There was significantly different among L5 and S1 group (*P* < 0.05).

**FIGURE 4:**
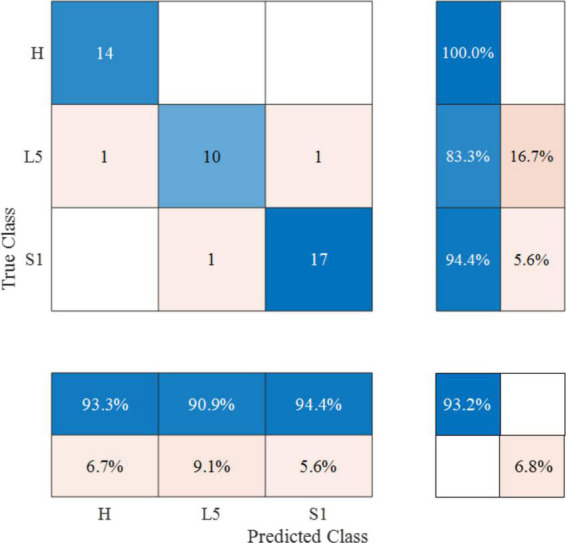
Confusion matrix of optimal RF diagnosis model based on SEMG parameters. The color scheme represents the consistency between the predicted and actual results. The numbers in the matrix denote the count of correctly predicted samples within specific categories. The percentages indicate the proportion of correctly predicted samples within those categories.

**FIGURE 5:**
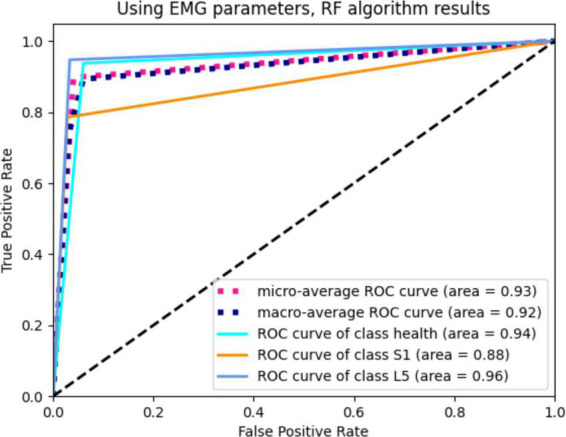
The ROC curve of RF diagnosis model based on SEMG parameters.

After 10 iterations of retention experiments, we confirmed that the diagnostic accuracy of the RF model based on the SEMG parameters was 84%. Additionally, the precision, recall, F1-score, and kappa values were found to be 85%, 84%, 0.84, and 0.76, respectively. The area under the ROC curve was calculated to be 0.93 (Figures 4, 5).

**FIGURE 6:**
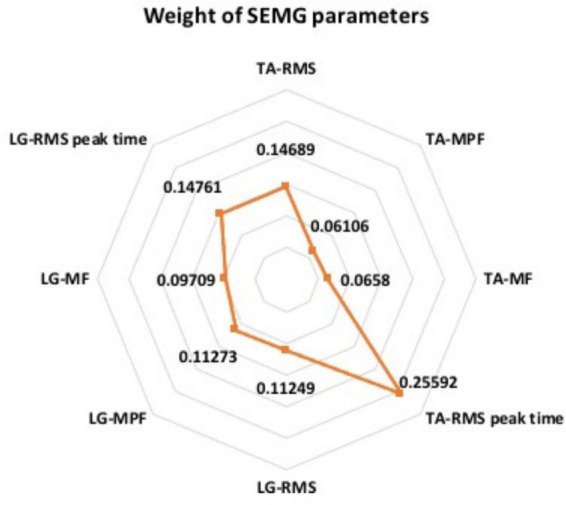
The weight of SEMG parameters in RF model.

Furthermore, we analyzed the weights of the RF model and found that among the eight SEMG parameters used in the model, the weights ranged from 6 to 26% (Figure 6). Notably, the RMS-peak time of TA had the highest weight (26%), followed by LG’s RMS-peak time (15% each).

## 4. Discussion

### 4.1. Analysis of SEMG characteristics in lower limb muscles

Lumbar disc herniation patients often experience reduced muscle strength and endurance. Previous studies have demonstrated that compression of the L5 nerve root can result in TA dysfunction, while compression of the S1 nerve root can lead to gastrocnemius dysfunction (Barr, 2013; Wang and Nataraj, 2014). Additionally, LDH patients with low back pain tend to experience increased multifidus muscle fatigue (Ramos et al., 2016). However, it can be challenging to diagnose the cause of abnormal gait when multiple pathological conditions coexist. In one case, peroneal nerve compression caused by ganglion cyst combined with L5 radiculopathy was observed, and electrical diagnosis was found to improve diagnostic accuracy in addition to MRI and other imaging methods (Park et al., 2019). SEMG is an effective tool for accurately assessing neuromuscular function in patients and has been widely used for clinical diagnosis and evaluation of various diseases and dysfunctions. While some studies have used SEMG to observe and record muscle function in LDH patients (Rønager et al., 1989; Dedering, 2012; Supuk et al., 2014; Djordjevic et al., 2015), most of these studies have focused on SEMG signals from paravertebral muscles and other muscles in the lumbar region, with only a few studies examining changes and dynamic adjustments in lower limb muscles in LDH patients. A study in 2020 found that abnormal gait in LDH patients was associated with abnormal lower limb muscle activity and neurological control disorders (Wang et al., 2020).

In this study, SEMG analysis was conducted on LDH patients, which revealed significant changes in the muscle activation patterns of patients with L5 and S1 nerve root compression. In cases where the L5 nerve root was compressed, neural control disorders were observed mainly in the TA. Under normal circumstances, the activation of TA muscle occurs prior to the completion of 12% of the gait cycle. However, in L5 group patients, the RMS-peak time of the TA in the symptomatic side was significantly delayed, with a median activation time of 35% of the gait cycle, resulting in a tendency of co-contraction with LG. Additionally, the MPF and MF were significantly decreased, and the RMS-peak of LG in the symptomatic side was also decreased. In cases where the S1 nerve root was compressed, neural control disorders were observed mainly in the LG. Normally, the activation peak of LG appears in the late stance phase of the gait cycle, but in S1 group patients, the activation time of LG was advanced, with the median activation time shifting from 44 to 27% in the symptomatic side, resulting in a bimodal activation pattern and a tendency of co-contraction with TA. The RMS-peak, MPF, and MF of LG decreased, and the RMS-peak of TA also decreased accordingly. Compared with the healthy group, LDH patients showed similar changes in muscle activation patterns on the symptomatic side. Moreover, compared with the S1 group and healthy group, the activation time of TA in the L5 group was significantly delayed, and the degree of fatigue in the TA was increased. The RMS-peak in LG was also significantly lower than in the S1 group. The RMS-peak time of LG in the S1 group was significantly advanced compared to that in the L5 group, and the MPF and MF of TA, and the RMS-peak and MPF of LG were significantly lower than in the healthy group.

The results of this study demonstrate that the functional state of the main muscles innervated by the corresponding nerve root changed when patients with different nerve root compressions were walking, which was related to neuromuscular control disorders after nerve compression (Li et al., 2018). This led to abnormal recruitment and fatigue of the corresponding muscle at a specific stage of the gait cycle. In the case of L5 nerve root compression, mechanical compression of the nerve can trigger conduction function abnormalities, resulting in a significant delay in the peak activation of the symptomatic TA and a significant increase in the overlap contraction area with LG. At this point, the LG and TA, a pair of antagonist muscles, exhibit a co-contraction phenomenon, which is an ineffective muscle coordination strategy that is different from normal alternating contraction. This finding is consistent with the findings of Wang et al. (2020), who also observed inappropriate co-contraction between the TA and gastrocnemius during walking in LDH patients.

Taking into account that LG is mainly innervated by the S1 nerve root, the compression of the L5 nerve root has a relatively small effect on LG. Therefore, to avoid dysfunction caused by muscle co-contraction, the RMS-peak of LG on the symptomatic side decreased accordingly. Co-contraction of antagonist muscles can cause joint stiffness or postural abnormalities (Lo et al., 2017; Du et al., 2018), significantly increasing energy expenditure during exercise and making muscles more prone to fatigue (Hallal et al., 2013). This is consistent with the decrease in MPF and MF of the symptomatic TA (Li et al., 2018). It may also partially explain why LDH patients often experience symptoms such as joint stiffness, claudication, muscle pain, and discomfort during walking (Wang et al., 2020).

When the S1 nerve root is compressed, the RMS distribution of LG changes to a bimodal activation pattern in the gait cycle, with the first peak occurring in the mid-stance stage. This early contraction of LG is thought to be a compensatory mechanism that helps speed up the transfer of the center of gravity, reduce weight-bearing on the symptomatic side, facilitate knee flexion, and reduce the length of the lower limb to avoid pain during single leg support. Our previous study also found similar SEMG changes in patients with S1 nerve root compression (Qie et al., 2020). Due to the compensatory contraction being small, the RMS-peak of LG was significantly lower on the symptomatic side than the asymptomatic side, and the earlier activation also led to an increase in the overlap contraction area with TA, which resulted in the same co-contraction of the antagonist muscles seen in L5 nerve root compression. Additionally, the RMS-peak of TA also decreased accordingly.

However, we also recognize that different diagnoses of L5 and S1 nerve root compression may lead to different treatment options. For example, L5 nerve root compression may result in altered activity patterns in the TA and may require physiotherapy targeting the TA to improve its function and reduce co-contraction with the LG. Conversely, when the S1 nerve root is compressed, the activity pattern of the LG is altered and physiotherapy targeting the LG may be required to improve its function. In forthcoming research endeavors, our aspiration is not only to delve deeper into this salient issue to assist clinicians in diagnosing the location of nerve root compression more accurately but also to evaluate pre- and post-operative EMG patterns. In particular, we are interested in scrutinizing cases that result in substantial nerve decompression and meaningful pain improvement post-surgery. Such assessments could serve as pivotal indicators of normalized EMG activation, as per our hypothesis, ultimately enhancing the scope and efficacy of treatment options for patients.

### 4.2. RF diagnosis model based on SEMG parameters

Random forest is a highly flexible and innovative machine learning algorithm that has a broad range of potential applications. It was proposed by American scholar Breiman in 2001, building on the classification tree algorithm developed in the 1980s (Breiman, 2001). Compared to other current algorithms, RF offers exceptional accuracy, can effectively handle large datasets, and is adept at processing input samples with high-dimensional features while evaluating the importance of each feature in classification problems. He et al. (2020) applied the RF algorithm to evaluate the gait of elderly individuals, demonstrating that RF improved gait classification accuracy. Shi et al. (2020) found that the RF algorithm can provide a solution for fusing human and exoskeleton equipment by giving corresponding weight to the original data, enhancing the real-time classification of traditional SEMG signals.

In this study, we trained an RF classification model using SEMG parameters. The training process involved ten repeated cross-validation experiments, where the model was trained on 50% of the patients and validated on the remaining 50% each time. The results showed that the RF model had a high diagnostic accuracy and could assist in localizing compressed nerve roots in LDH patients. We used the area under the ROC curve as the performance metric to evaluate the model’s performance. The ROC curve is a plot of sensitivity (true positive rate) against 1-specificity (false positive rate) for different threshold values. A larger area under the curve indicates higher diagnostic accuracy. Based on our results, the RF model achieved an area under the ROC curve of 0.93, which indicates that it is an effective diagnostic model.

In addition, RF algorithm can give corresponding weights to the original data, score the classification ability of different parameters, and identify the parameters that play an important role in the classification. Based on the characteristics of RF, we also compared the parameter weights of this model, and found that the RMS-peak time of TA has the highest weight ratio (26%). It is suggested that RMS-peak time of TA can be used as the most important SEMG parameter to identify L5 or S1 compressed nerve roots, which can provide further reference for optimizing the diagnosis model in the future.

Our study is a replication of the observational study by Li et al. (2018) and we are in a larger, new cohort of patients where we confirm previous findings and also further expand the knowledge in this area. Our findings suggest that the RF model can assist in the localization and diagnosis of compressed nerve roots in LDH patients, while the SEMG parameters can provide a further reference for optimizing the diagnostic model.

However, while SEMG is a powerful tool to help us understand the mechanisms of disease, the feasibility of implementing such a diagnostic procedure in a clinical setting also needs to be considered. While SEMG equipment is relatively easy to obtain and use, motion capture systems may require more equipment and space. In addition, some training and equipment maintenance may be required in order to integrate such a diagnostic procedure with existing diagnostic processes.

Despite these challenges, we believe that as technology advances and costs decrease, the use of SEMG and motion capture systems in clinical settings will become increasingly feasible. We look forward to future research that will further explore the implementation of such diagnostic procedures to help clinicians more accurately diagnose the location of nerve root compression and provide better treatment options for patients.

## 5. Conclusion

This study highlights the potential of SEMG as a diagnostic tool for LDH patients with L5 and S1 nerve root compression. The differences in SEMG characteristics between TA and LG during walking provide valuable insights into the location of nerve root compression. The RF algorithm-based diagnostic model demonstrated high accuracy, precision, and recall, indicating its potential as a reliable diagnostic tool. The model’s ability to identify the weights of different SEMG parameters provides clinicians with a better understanding of the relative importance of each parameter in diagnosis.

Overall, this study suggests that SEMG can serve as an effective complementary diagnostic tool for LDH, helping clinicians accurately diagnose the location of nerve root compression and provide better treatment options for patients.

## Data availability statement

The raw data supporting the conclusions of this article will be made available by the authors, without undue reservation.

## Ethics statement

The studies involving human participants were reviewed and approved by the Ethics Committee of Capital Medical University, Beijing, China. The patients/participants provided their written informed consent to participate in this study.

## Author contributions

YW, HW, and SQ contributed to the concept and design of the study. YL organized the database. YW and HW performed the statistical analysis and wrote the first draft of the manuscript. YW, HW, YL, and CW wrote parts of the manuscript. All authors contributed to revision, read, and approved the submitted version of the manuscript.

## Footnotes

1
https://www.psychologie.hhu.de/arbeitsgruppen/allgemeine-psychologie-und-arbeitspsychologie/gpower.html

2
http://www.seniam.org
